# Evaluation of 360° Image Projection Formats; Comparing Format Conversion Distortion Using Objective Quality Metrics

**DOI:** 10.3390/jimaging7080137

**Published:** 2021-08-05

**Authors:** Ikram Hussain, Oh-Jin Kwon

**Affiliations:** Department of Electrical Engineering, Sejong University, 209 Neungdong-ro, Seoul 05006, Korea; contactik09@gmail.com

**Keywords:** 360° image, image quality evaluation, objective image quality metric, cube-map projection, cylindrical projection, equi-rectangular projection, equal-area projection

## Abstract

Currently available 360° cameras normally capture several images covering a scene in all directions around a shooting point. The captured images are spherical in nature and are mapped to a two-dimensional plane using various projection methods. Many projection formats have been proposed for 360° videos. However, standards for a quality assessment of 360° images are limited. In this paper, various projection formats are compared to explore the problem of distortion caused by a mapping operation, which has been a considerable challenge in recent approaches. The performances of various projection formats, including equi-rectangular, equal-area, cylindrical, cube-map, and their modified versions, are evaluated based on the conversion causing the least amount of distortion when the format is changed. The evaluation is conducted using sample images selected based on several attributes that determine the perceptual image quality. The evaluation results based on the objective quality metrics have proved that the hybrid equi-angular cube-map format is the most appropriate solution as a common format in 360° image services for where format conversions are frequently demanded. This study presents findings ranking these formats that are useful for identifying the best image format for a future standard.

## 1. Introduction

In various applications, including social networking services, teleconferencing, broadcasting, map services, education, professional training, and online games, 360° immersive content has recently become popular. Unlike traditional video, virtual reality (VR) provides 360° content, that is, a bounding sphere containing the entire scene with 360° horizontal and 180° vertical directions to provide a rich user experience. In addition, VR applications pursuing real-life simulations on advanced digital devices have been an increasingly intriguing topic [1,2,3,4,5,6,7,8,9]. VR services are rapidly growing in number, providing high-quality 360° content. Accordingly, the demand for 360° content has proliferated with increased attention; however, it should be noted that there are still numerous impediments for 360° image processing. Furthermore, head-mounted devices such as HTC Vive, Samsung Gear VR, and Oculus Rift allow users to change their point of view and dynamically view 360° content. Users can simply select the content by changing the viewing direction in a head-mounted display.

For an immersive visual experience, a higher resolution and frame rate (e.g., 8K at 90 fps) are expected. Therefore, the file size of the 360° content tends to be extremely large; this requires extensive resources for storage and bandwidth and causes transmission delays. Therefore, improving the compression efficiency of 360° content is in urgent demand [10]. Thus far, conventional image and video codings, such as JPEG image and high-efficiency video coding [11], have been used for the compression of spherical domain images and videos, significantly affecting the development of VR applications.

The joint video exploration team (JVET) from ITU-T VCEG (Q6/16) and ISO/IEC MPEG (JTC1/SC29/WG11) was established in October 2015 to study the potential requirements for a future video coding standard, including 360° video coding technologies, the application requirements, and the delivery aspects [12]. The committee began the standardization of next-generation video compression standards, called Versatile Video Coding (VVC). VVC will support projection formats that achieve better coding performance and additional features to enhance such a performance, including packing methods. Optimal projection formats for 360° content are being explored by MPEG and JPEG to enhance coding efficiency. In these two organizations, an image coding standard for 360° images has yet to be established. However, a next-generation image coding standard, called JPEG XL, is being standardized, including the use of 360° image coding for augmented/virtual reality.

Because the observation space of 360° content is a sphere, it is not easy to represent or process digital content. Considering the development of 360° content and the efficiency of current conventional video coding standards, projecting the original spherical image into a two-dimensional (2D) plane for encoding is a common choice, as suggested by JVET, allowing current video coding frameworks to be used. Projection techniques for 360° images and videos have become a fundamental part of the spherical image and video coding.

Various projection formats are now available for 360° images and videos. However, the transformation from a sphere into a 2D plane introduces several artifacts, such as sample redundancy, discontinuous boundaries, and shape distortion [13]. Redundancy in a sample causes many invalid pixels to be coded. Irregular boundaries affect the prediction performance, and a shape distortion leads to inefficiency in the coding performance.

In general, different projection methods cause different types of artifacts. As an example, an equi-rectangular projection (ERP) suffers from redundant samples and a horizontal stretching problem near the pole areas. Another example is the texel (the smallest unit of texture) area distortion introduced in spherical cube-maps [14]. It has been reported that a spherical surface cannot be projected onto a 2D plane without distortion, because the projection is an irreversible nonlinear process. If a projection preserves its shape, it does not preserve its area, and vice versa.

For research on the design of highly efficient 360° image and video services, and to better explore the use of resources and achieve better quality as perceived by users, it is necessary to evaluate the performance of various projection formats, which have inherent effects on the 2D plane. Many projection formats have been proposed thus far, and most have their own specific merits. The analysis and quality assessment of the various projection formats currently used on the market are useful to industry players that want to provide 360° VR services effectively and reliably.

It is currently common for 360° image services to support multiple projection formats and provide users with a personalized and immersive experience through the internet with higher quality and low latency. More than 40 360° image services have been surveyed to see which projection format is mainly considered and how many formats they use. It was observed that the support of multiple projection formats is common in the industry, based on user interest. The user can change the projection format of the content for a more personalized experience.

Each format has its own merits and demerits, and the contents differ from conventional 2D images. A 2D image is generated by unfolding the pixel information from the spherical space of 360° content to the 2D plane. On the display side, an inverse projection is applied to map the image back to the sphere for representing 360° content. Owing to the aforementioned industrial needs, the ISO/IEC JTC1 SC29/WG11, Moving Picture Experts Group (MPEG) developed the Omnidirectional MediA Format (OMAF) standard, which defines the format of the media application, supporting 360° omnidirectional video services. OMAF currently supports two projection formats: ERP and cube-map [15]. Although the standardizations mentioned above are focused on different aspects, a common and essential consideration is the definition of the projection format, which can be used in various applications as a default.

As stated previously, 360° images can be projected using various methods, and JVET has been exploring 360° video coding and processing, supporting state-of-the-art projection formats. JVET recruited participants from the industry who have proposed many projection formats [15] for future standards. The projection formats selected for evaluation in this study are mostly based on their results. ERP is the most widely used projection format in the industry for viewing 360° panoramas, because it is intuitive and easy to generate [16,17,18]. Besides, ERP has only one face, which is easy to visualize. However, it suffers from severe stretching at the north and south poles of the sphere, which in turn reduces the coding efficiency and increases the bandwidth consumption. Other basic projection formats used for 360° images include an equal-area projection (EAP) [18,19], cube-map projection (CMP) [20,21,22], and cylindrical projection (CP). CP is a perspective projection that preserves the scale of vertical objects, for example, buildings, which is imperative for architectural scenes.

CMP has three modified projection formats: an adjusted cube-map projection (ACP) [23], an equi-angular cube-map projection (EAC) [24,25], and a hybrid equi-angular cube map projection (HEC). It was recently reported in JVET that HEC achieves a better coding efficiency [26], because it has a more uniform sampling distribution [27]. EAP and CP also have a modified projection, i.e., adjusted equal-area projection (AEP) and equatorial cylindrical projection (ECP), respectively. ECP is a modification of a Lambert cylindrical equal-area projection [28]. The sphere is partitioned into three regions: equatorial, north-pole, and south-pole regions. The equatorial region of the sphere is projected using the Lambert cylindrical equal-area projection, and the pole regions are projected onto the squares. Pyramid mapping was also introduced. The base of the pyramid contains the full resolution, whereas the remaining parts are mapped with decreasing resolution toward the sides [29].

**Figure 1:**
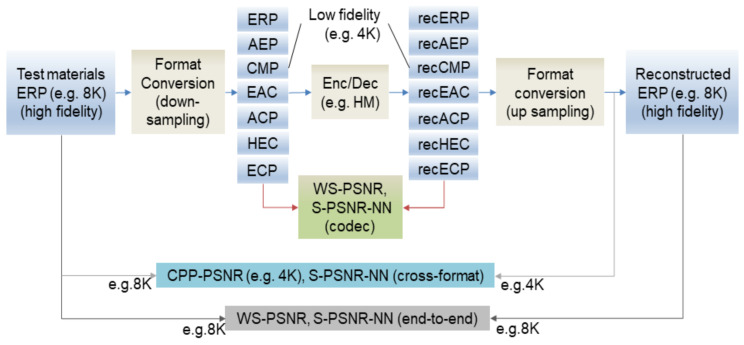
Pipeline for evaluating 360-degree video coding as recommended by JVET [30].

The pipeline used for evaluating the 360° video coding of JVET is shown in Figure 1 [30]. It focused on the compression performance of different 360° video projections. First, high-fidelity test materials are provided in ERP format, which is regarded as the ground truth for quality evaluation. Various format conversions are applied to these materials for a comparison of the coding efficiency. For quality evaluation, uniform quality evaluation methods in a spherical domain, such as the Craster parabolic projection peak signal-to-noise ratio (CPP-PSNR), spherical PSNR (S-PSNR), and weighted-to-spherical PSNR (WS-PSNR), are selected. This testing procedure [30] has mainly been designed for exploring the dependency of the coding efficiency on different projections and for defining the quality evaluation metrics.

An evaluation of the coding efficiency of different projections for 360° videos, their comparison, and the evaluation criteria are reported in [31]. The authors followed the JVET pipeline and used quality metrics designed for 360° viewing. They reviewed the projection formats and quality evaluation methods, among which various orientations of 360° video were studied. A study on the performance of objective quality metrics for 360° visual content was also conducted [32], and the authors concluded that the objective quality metrics designed for 360° content do not outperform the conventional metrics designed for 2D images. In addition, Xiu et al. [33] reported that the ERP projection format has an obvious edge over other projection formats for video sequences, whereas all formats other than ERP suffer from a format conversion loss. The projection formats of ERP, CMP, AEP, and ACP are preferentially referenced in [31].

These formats unnecessarily oversample in certain regions and have been criticized for wasting bits in their encoding. Thus, a variety of mapping methods with less distortion and pixel wasting have been proposed [34,35,36,37]. Although a tremendous number of studies have been conducted for 360° videos [38,39,40], the standardization activities for a quality assessment of 360° images are limited.

In this study, the performances of state-of-the-art projection formats for 360° images are evaluated. The aim is to find the projection format that causes the least distortion when one projection format is converted into another. Many projection formats have been proposed thus far; however, additional data for ranking these projection formats are needed. All formats excluding ERP need to go through one extra format conversion step (that is, conversion from their native format into the coding format). The format conversion step always introduces loss; the native format has a favorable bias, because it does not suffer from such loss. Thus, for 360° image services that require format conversion for coding efficiency, it would be desirable to propose a single common projection format as the most appropriate format among all available state-of-the-art projection formats.

The main contributions of this study can be summarized as follows:

- A framework that measures the distortion between different projection formats is proposed without bias toward any projection format.
- The most recent projection formats are included in the evaluation.
- Both conventional and advanced metrics are used for a quality assessment.
- The evaluation focuses on finding the highest-ranked projection format and comparing the projection formats with the ERP and with each other based on three frequently used image sizes.

The rest of this paper is organized as follows. Section 2 presents the proposed method for an evaluation of 360° image projection formats. Section 3 presents the experimental setup, a methodology for selecting the test datasets, and the evaluation results. Finally, Section 4 concludes the paper with directions for future research.

## 2. Proposed Method

**Figure 2:**
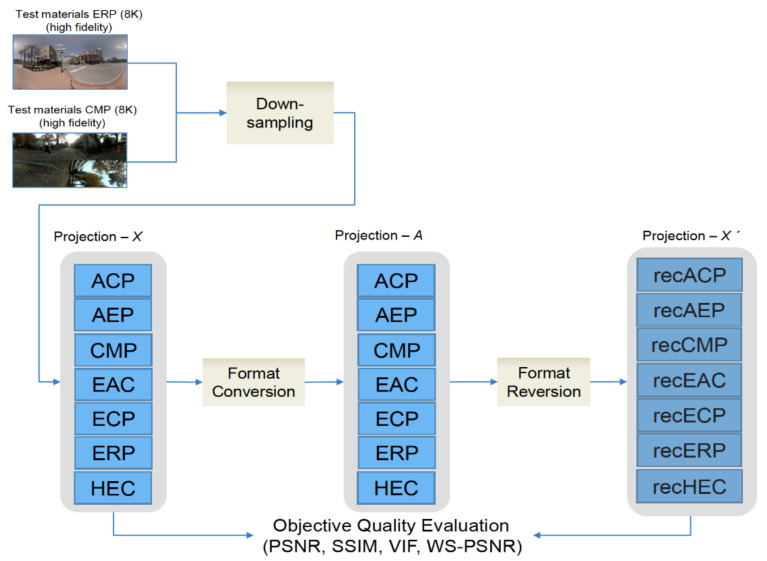
Proposed framework for the evaluation of projection formats. Projection–*A* can be any projection except Projection–*X*. Projection–*X*’ means reconverted projection format of Projection–*X* from Projection–*A*.

Figure 2 shows the proposed framework for evaluating the distortions caused by projection format conversion. Seven projection formats, i.e., ACP, AEP, CMP, EAC, ECP, ERP, and HEC, were chosen for the evaluation. Such variety has confused the industry and researchers in selecting a high-quality format for their various approaches. Therefore, it is necessary to produce data when choosing a high-quality projection format for future 360° image standards.

The goal is to propose a projection format that shows the least distortion during a format conversion, allowing it to be used as the most appropriate common projection format for 360° image services in which format conversions are frequently demanded. As discussed in Section 1, previous studies focusing on coding were conducted by JVET. Although format conversion is frequently demanded in the market, the loss of content from a format conversion has yet to be analyzed. The proposed framework was, therefore, specifically designed to measure the format conversion loss before coding the content without bias to any of the formats. The framework eliminates bias by using all formats as the starting point of the framework, whereas traditional quality evaluation frameworks use the ERP format as the starting point.

For a fair evaluation, the original 360° image of Projection–*X* (for all projection formats) is generated, without bias toward the native projection format (ERP) when comparing the performance of different projection formats and their effects [33]. Projection–*X* is generated by two sources, i.e., 8K ERP and CMP images. The down-sampling process is used to reduce unfair bias among ERP and other projections [30]. The evaluation conducted to achieve the purpose of this paper, which is finding the most appropriate projection format, under the proposed procedure as follows:

(1) Generate the original 360° image (Projection–*X*) by down-sampling the high-fidelity test image.
(2) Convert the image (Projection–*X*) into another format, denoted by Projection–*A*, excluding the original format of Projection–*X*.
(3) Convert the image (Projection–*A*) back into the format of the original Projection–*X*.
(4) Calculate the distortion between the original (Projection–*X*) and the reconverted images (Projection–*X*’) using the objective quality assessment.
(5) Repeat steps 1 through 4 by changing the format of Projection–*A* for all formats under evaluation except Projection–*X* itself for each Projection–*X*. A format showing the least distortion is found when a given format (Projection–*X*) is converted into another format (Projection–*A*) and reconverted into the given format.
(6) Repeat step 5 by changing the format of Projection–*X* for all formats under evaluation and calculating the average distortion for each Projection–*A*.
(7) The projection format showing the least average distortion is proposed based on the results of 6.

Projection–*X* format images were generated in three sizes, 1920 × 960, 3840 × 2560, and 5760 × 2880, denoted by 2K, 4K, and 6K, respectively, as recommended by JVET [41], and used as the original image for measuring the distortions.

## 3. Experimental Results

In this section, a detailed description of the experiments under the proposed evaluation framework in Section 2 is discussed. The software used for format conversion is introduced, and the selection of quality metrics and datasets is described and presented. Finally, an analysis of the results is presented, followed by a discussion. The primary purpose of the experiment is to evaluate the overall quality of each 360° projection format compared with the other seven selected formats.

### 3.1. Experimental Setup

This paper evaluates the distortion caused by the format conversion from one of the seven formats into another. For the conversion of the projection formats, 360Lib software (version 8.0) [42], developed by JVET for the use of a future standardization, was applied. The library supports conversions between various projection formats, including ACP, AEP, CMP, EAC, ECP, ERP, and HEC. It can also change the image size of the converted image, including the frame packing configurations for the CMP. For the experiment, original images of Projection–*X* were first prepared by converting the test materials. Projection–*X* is converted into Projection–*A* and then reconverted into Projection–*X* using the 360Lib software to evaluate the distortion owing to the format conversion.

A subjective quality assessment is more favorable for measuring the image quality; however, an objective quality assessment is a fast and reliable method to assess the image quality if effective metrics exist. For objective evaluation, the PSNR, structural similarity index metric (SSIM) [43], visual information fidelity (VIF) [44], and WS-PSNR measures were adopted to compare the original and the reconstructed images after conducting the format conversions. The PSNR was chosen as the most popular objective image quality metric, and previous studies on an objective assessment [45] have reported that PSNR-related quality measures are well correlated with a subjective quality assessment when evaluating 360° images. WS-PSNR [46,47], which was proposed by experts to improve a conventional PSNR for 360° images, was also chosen. It has been claimed that the uniformly weighted calculation adopted in the PSNR cannot provide the correct measurement of the objective quality for 360° images. To correctly measure the distortion in the observation space, the quality should be evaluated spherically. Therefore, with WS-PSNR, each pixel’s error on the projection plane is multiplied by the weight to ensure that the equivalent spherical area in the observation space has the same influence on the distortion measurements. The weights of the ERP and CMP formats are described in [47].

To grant a critical aspect of human perception of the spatial relationship between pixels, SSIM and VIF are used to measure the structural degradation and fidelity of the image, respectively. Furthermore, Upenik et al. [32] reported that metrics designed for 360° content do not outperform the conventional metrics designed for 2D images, and thus both advanced and conventional metrics were used. It should be noted that the evaluation using additional measures, i.e., S-PSNR [48], CPP-PSNR [49], WSNR, SNR, and MSE, are not included in this study, because their results yielded the same conclusion.

**Figure 3:**
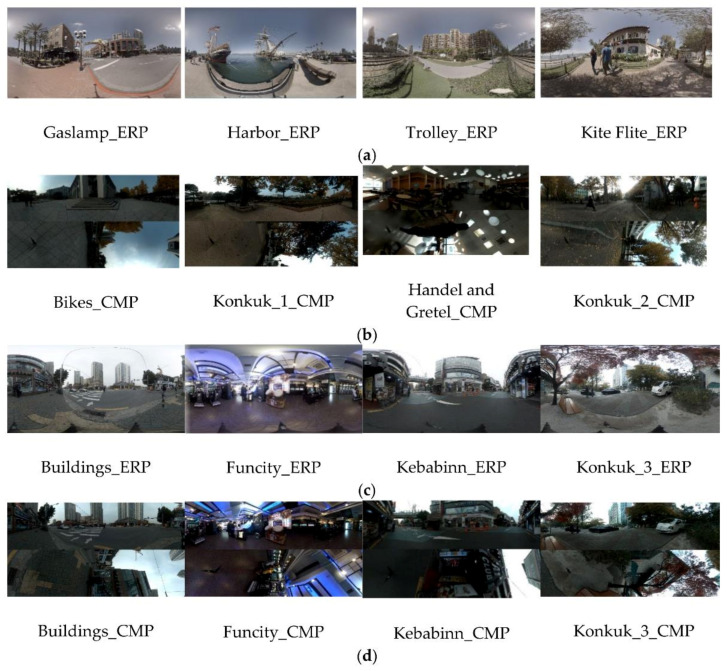
Selected test materials: (**a**) 8K ERP with size 8192 × 4096 [50], (**b**) 8K CMP with size 7680 × 3840, (**c**) 8K ERP with size 7920 × 5280, and (**d**) 8K CMP with size 7920 × 5280.

Figure 3 shows the test materials comprising eight 8K equi-rectangular panoramic images (8192 × 4096 or 7680 × 3840) and eight 8K cube-map images (7920 × 5280). Figure 3a shows sample images provided in 8K ERP format using InterDigital [50]. Figure 3b–d show the sample images captured by authors through Insta360 Pro with six 8K lenses. Figure 3b shows the 8K CMP images obtained from the six raw images using Panorama10 [51]. Figure 3c shows the images in the 8K ERP format, whereas Figure 3d shows the same scene as in Figure 3c in 8K CMP format achieved by Panorama10. All sample images are in the form of a raw YUV format and consist of various scenes, including landscapes; architecture; and indoor, outdoor, and night scenes.

**Figure 4:**
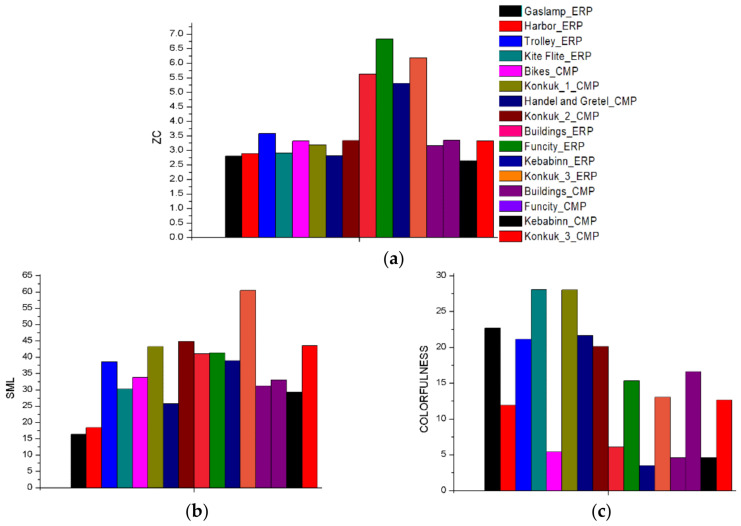
Attribute distribution of datasets: (**a**) ZC, (**b**) SML, and (**c**) colorfulness.

Sample images were chosen to include a variety of image quality attributes. In previous studies on the human perception of image quality, several attributes have been proposed for an image quality assessment, for example, the overall luminance, contrast, sharpness, details, naturalness, and colorfulness [52,53,54,55,56,57,58]. In this study, three metrics, i.e., zero-crossing (ZC), sum-modified Laplacian (SML), and colorfulness, are adopted as the major representative metrics owing to their simplicity and fast calculation properties, and thus the diversity of the sample images may easily be verified. For the definitions of ZC, SML, and colorfulness, refer to [59]. Initially, more than 80 images were collected, and 16 images were then selected to cover a variety of these metric values. Figure 4 shows the graphical values of ZC, SML, and the colorfulness of the selected sample images.

### 3.2. Experimental Results and Discussion

**Figure 5:**
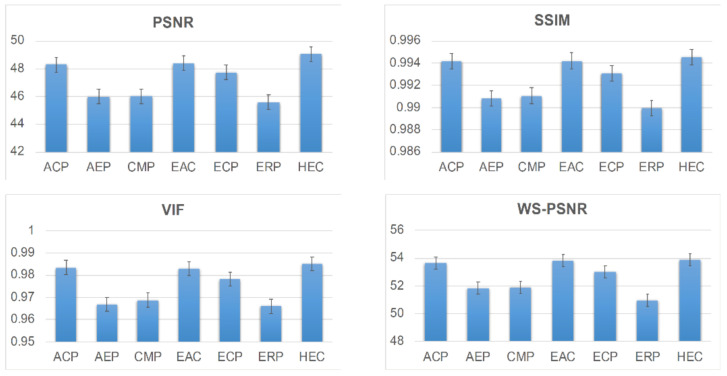
Overall evaluation of projection formats using PSNR, SSIM, VIF, and WS-PSNR.

Table 1, Table 2, Table 3 and Table 4 provide averaged distortion values over the three image sizes (2K, 4K, and 6K) for each distortion metric resulting from step 6 in Section 2. When ACP, CMP, EAC, or ECP are used for Projection–*X*, the results of the HEC are the highest for the PSNR, SSIM, and VIF metrics. AEP is a modified version of ERP. When Projection–*X* is AEP and ERP, ERP and AEP showed the best results, and HEC showed the second-best results. In the case of the WS-PSNR shown in Table 4, the individual ranking is different from the other three metrics, except for the ERP and ECP formats. Nevertheless, it can be seen that the overall results are comparable with those of the other three metrics, as shown in Figure 5. The results of the evaluation can be summarized as follows:

(1) When focusing on the final decision on the highest-ranking projection format, it can be concluded that the dependency of the evaluation results on the quality metric used and on the size of the image is almost negligible.
(2) For most cases, ACP and EAC, which are modified versions of CMP, showed compatible results.
(3) It should also be noted that no significant difference was found when 8K CMP or ERP is employed as the source image.
(4) From the overall evaluation, HEC is recommended as a common projection format when the format conversion distortion measured by the objective metrics is considered.

## 4. Conclusions and Future Studies

In this study, we presented an overview of the 360° image projection formats, and seven state-of-the-art 360° image projection formats, ACP, AEP, CMP, EAC, ECP, ERP, and HEC, were evaluated. While previous works for evaluating 360° image or video projection formats were performed in the coding efficiency point of view [41,60], our evaluation is based on the format conversion distortion perspective employing four objective metrics: PSNR, SSIM, VIF, and WS-PSNR. Based on three image quality attributes, i.e., ZC, SML, and colorfulness, 16 among more than 80 images were selected as the source images for the evaluation.

We proposed an evaluation framework specifically designed to measure the format conversion distortion. The proposed framework eliminates bias by using all formats as the starting point of the framework, whereas traditional quality evaluation frameworks use the ERP format as the starting point.

It was concluded that ERP, which is a mainly used format in the industry, is not an appropriate projection format in terms of projection format conversion distortion. HEC has been found to be the most acceptable and is recommended as the best common projection format among the state-of-the-art projection formats for 360° image services, where projection format conversions are frequently required.

However, we authors would like to note that all the projection formats considered in this paper always introduce their own structural distortions. Based on the best of our knowledge, we have chosen four metrics: PSNR, SSIM, VIF, and WS-PSNR, for our evaluation and introduced the reason why we chose them in Section 3.1. Unfortunately, these current state-of-the-art metrics can be claimed to not measure structural distortions fully. We think that it has to be mentioned here that further research on human psychophysical evaluations and new perceptual metrics specialized on 360° images will make our results more complete.

The evaluation described herein was conducted for 360° images from a format conversion perspective, and future research extending the evaluation for the 360° video sequences should prove interesting. It may be also valuable future research for the community to carry out a more comprehensive set of subjective tests using state-of-the-art image coding algorithms. As for additional future research topic, it would be also interesting to investigate the effects of various viewing devices such as head-mounted device, mobile screen, TV, etc. on subjective evaluations of 360° image projection formats.

## Figures and Tables

**Table 1:** Comparison using PSNR (average values at 2K, 4K, and 6K).

Projection–*X*	Projection–*A*						
	ACP	AEP	CMP	EAC	ECP	ERP	HEC
ACP		45.19650	46.08579	48.55320	47.50620	44.72290	***49.25448***
AEP	47.89280		45.68754	47.79910	47.41540	***48.76410***	48.52101
CMP	48.11457	45.28200		48.08930	47.30470	44.78660	***48.92551***
EAC	48.56022	45.18870	46.08233		47.81450	44.74710	***49.58164***
ECP	47.83781	45.27810	45.82864	48.14880		44.72550	***49.30380***
ERP	47.91150	***48.80798***	45.38214	47.86570	47.20290		48.68465
HEC	49.49098	46.22400	47.04229	***49.81030***	49.16740	45.74050	
**Overall**	48.30131	45.99621	46.01812	48.37780	47.73520	45.58110	***49.04518***

**Table 2:** Comparison using SSIM (average values at 2K, 4K, and 6K).

Projection–*X*	Projection–*A*						
	ACP	AEP	CMP	EAC	ECP	ERP	HEC
ACP		0.990515	0.991396	0.994684	0.99319	0.98860	***0.995205***
AEP	0.99379		0.990420	0.993699	0.99300	***0.99542***	0.994191
CMP	0.99360	0.989145		0.993543	0.99219	0.98781	***0.994070***
EAC	0.99483	0.989877	0.991686		0.99356	0.98873	***0.995437***
ECP	0.99397	0.990192	0.991206	0.994261		0.98867	***0.994927***
ERP	0.99315	***0.993720***	0.988772	0.993053	0.99179		0.99356
HEC	0.99573	0.991551	0.992935	***0.995948***	0.99485	0.99037	
**Overall**	0.99418	0.990833	0.991069	0.994198	0.99309	0.98994	***0.99456***

**Table 3:** Comparison using VIF (Average values at 2K, 4K, and 6K).

Projection–*X*	Projection–*A*						
	ACP	AEP	CMP	EAC	ECP	ERP	HEC
ACP		0.96641	0.97541	0.99309	0.98665	0.96391	***0.99439***
AEP	0.97543		0.95389	0.97419	0.97082	***0.98649***	0.97726
CMP	0.98245	0.95643		0.98175	0.97462	0.95069	***0.98440***
EAC	0.99317	0.96642	0.97679		0.98720	0.96438	***0.99483***
ECP	0.98572	0.96565	0.97271	0.98596		0.96071	***0.98794***
ERP	0.96924	***0.97536***	0.94447	0.96771	0.96033		0.97162
HEC	0.99486	0.97189	0.99026	***0.99505***	0.98974	0.96993	
**Overall**	0.98348	0.96703	0.96892	0.98296	0.97823	0.96602	***0.98507***

**Table 4:** Comparison using WS-PSNR (average values at 2K, 4K, and 6K).

Projection–*X*	Projection–*A*						
	ACP	AEP	CMP	EAC	ECP	ERP	HEC
ACP		51.58774	52.10874	***54.55395***	53.27640	51.1952	53.35633
AEP	***53.95220***		52.25159	53.95116	53.92140	53.6263	53.83587
CMP	***53.76260***	51.65417		53.81399	53.14770	51.2239	53.63988
EAC	***54.45870***	51.53064	52.07229		53.51250	51.1536	54.29657
ECP	53.44990	51.55605	51.91927	53.68819		51.0428	***53.96543***
ERP	54.39310	***56.48944***	52.65166	54.34450	54.09750		54.31771
HEC	***52.02060***	48.26435	50.52969	52.48458	50.26680	47.5490	
**Overall**	53.67280	51.84706	51.92221	53.80606	53.03710	50.9651	***53.90196***

## Data Availability

Not applicable.
